# A mouse model displays host and bacterial strain differences in *Aerococcus urinae* urinary tract infection

**DOI:** 10.1242/bio.058931

**Published:** 2021-08-13

**Authors:** Nicole M. Gilbert, Brian Choi, Jingjie Du, Christina Collins, Amanda L. Lewis, Catherine Putonti, Alan J. Wolfe

**Affiliations:** 1 Department of Pediatrics, Division of Infectious Diseases, Washington University School of Medicine, St. Louis, MO 63110, USA; 2 Department of Microbiology and Immunology, Stritch School of Medicine, Loyola University Chicago, Maywood, IL 60153, USA; 3 Department of Obstetrics, Gynecology, and Reproductive Sciences, University of California San Diego School of Medicine, La Jolla, CA 92093, USA; 4 Bioinformatics Program, Loyola University Chicago, Chicago, IL 60660, USA; 5 Department of Biology, Loyola University Chicago, Chicago, IL 60660, USA

**Keywords:** Gram-positive, Inflammation, Kidney, Urinary tract infection

## Abstract

In recent years, the clinical significance of *Aerococcus urinae* has been increasingly recognized. *A. urinae* has been implicated in cases of urinary tract infection (UTI; acute cystitis and pyelonephritis) in both male and female patients, ranging from children to older adults. *Aerococcus urinae* can also be invasive, causing urosepsis, endocarditis, and musculoskeletal infections. Mechanisms of pathogenesis in *A. urinae* infections are poorly understood, largely due to the lack of an animal model system. In response to this gap, we developed a model of *A. urinae* urinary tract infection in mice. We compared *A. urinae* UTI in female C3H/HeN and C57BL/6 mice and compared four clinical isolates of *A. urinae* isolated from patients with UTI, urgency urinary incontinence, and overactive bladder. Our data demonstrate that host genetic background modulates *A. urinae* UTI. Female C57BL/6 female mice rapidly cleared the infection. Female C3H/HeN mice, which have inherent vesicoureteral reflux that flushes urine from the bladder up into the kidneys, were susceptible to prolonged bacteriuria. This result is consistent with the fact that *A. urinae* infections most frequently occur in patients with underlying urinary tract abnormalities or disorders that make them susceptible to bacterial infection. Unlike uropathogens such as *E. coli*, which cause infection and inflammation both of the bladder and kidneys in C3H/HeN mice, *A. urinae* displayed tropism for the kidney, persisting in kidney tissue even after clearance of bacteria from the bladder. *Aerococcus urinae* strains from different genetic clades displayed varying propensities to cause persistent kidney infection. *Aerococcus urinae* infected kidneys displayed histological inflammation, neutrophil recruitment and increased pro-inflammatory cytokines. These results set the stage for future research that interrogates host-pathogen interactions between *A. urinae* and the urinary tract.

## Abstract (precis)

**Summary:***Aerococcus urinae* clinical isolates are genetically diverse and display differential capacity to cause UTI in a mouse model. Infection was rapidly cleared from the bladder, but persisted and caused inflammation in the kidney.

## INTRODUCTION

*Aerococcus urinae* is a Gram-positive, alpha-hemolytic bacterium first identified as a cause of urinary tract infection (UTI) (Aguirre and Collins, 1992). Since its initial description, the reports of *A. urinae* infections have continued to rise. Awareness of *A. urinae* as an emerging pathogen increased, especially following the advent of MALDI-TOF mass spectrometry techniques that allowed the organism to be discerned from staphylococci and streptococci (Meletis et al., 2017; Cattoir et al., 2010). *Aerococcus urinae* infects both males and females (Schuur et al., 1997), especially those with local or systemic predisposing conditions, such as elderly patients (Senneby et al., 2015) and those with urologic conditions (Sierra-Hoffman et al., 2005), prostatic diseases (Shelton-Dodge et al., 2011), urologic cancer (Higgins and Garg, 2017) or using urinary catheters (Sierra-Hoffman et al., 2005; Yu et al., 2019). *Aerococcus urinae* infections have also been reported, albeit rarely, in younger and apparently otherwise healthy patients (Yabes et al., 2018; Sous et al., 2019) and pediatric cases are beginning to emerge (Sous et al., 2019; Qureshi and Patel, 2018). *Aerococcus urinae* has also been detected in patients without clinically-defined UTI (Sierra-Hoffman et al., 2005), and was more frequently detected in urine from postmenopausal women with urgency urinary incontinence (UUI) compared to asymptomatic controls (74% UUI, 28% non-UUI; *P*=0.002; *n*=118) (Pearce et al., 2014). In addition to colonization and infection of the urinary tract, *A. urinae* can cause invasive infections like bacteremia (Senneby et al., 2016), urosepsis (Kim et al., 2017), endocarditis (Tai et al., 2021; Ludhwani et al., 2020), aortitis (Chhibber et al., 2019), and spondylodiscitis (Rougier et al., 2018; Lyagoubi et al., 2020), as well as other musculoskeletal infections (Greco et al., 2018) and soft tissue infections (Forsvall et al., 2019). The urinary tract is often implicated as the source of these disseminated infections because *A. urinae* is concurrently detected in urine (Senneby et al., 2016; Lyagoubi et al., 2020).

The first complete genome sequence of *A. urinae* was reported in 2016 for the urinary isolate CCUG 36881T from a case of UTI (Carkaci et al., 2016). Whole genome sequencing and analysis of an additional 40 *A. urinae* isolates from infection episodes of UTI, bacteremia and infective endocarditis (IE) revealed substantial genetic diversity with respect to genome size, the number and identity of core genes and the presence of putative capsular polysaccharide loci (Carkaci et al., 2017). There was also variability among *A. urinae* genomes regarding the presence of homologs of known virulence factors, including genes predicted to mediate bacterial adherence and protect against phagocytosis. One hypothesis to explain the wide range of clinical conditions associated with *A. urinae* is that different strains contain the genetic makeup necessary to cause particular phenotypes. However, in a previous study, there was no apparent phylogenetic distinction between strains isolated from urine or blood, or between those from patients with UTI or bacteremia (Carkaci et al., 2017). We recently reported the genome sequences of 24 strains obtained by transurethral catheterization of 21 women with and without lower urinary tract symptoms, including those associated with UUI, stress urinary incontinence (SUI), overactive bladder (OAB) and UTI. We found that their ability to self-aggregate (i.e. flocculate) was related to phylogeny (Hilt et al., 2020). However, whether or not there are differences between *A. urinae* strains isolated from infection cases (e.g. UTI, bacteremia, endocarditis) versus other conditions associated with lower urinary tract symptoms (e.g. UUI, OAB) has not been examined at the level of genomics or in experimental systems.

Since the clinical importance of *A. urinae* has been recognized only relatively recently, we know little about how the bacterium colonizes or infects the urinary tract and disseminates to cause disease in other body sites. Likewise, we do not know what host responses may mitigate or exacerbate *A. urinae* infection. To begin to address these gaps in knowledge, we developed a model of *A. urinae* UTI in female mice. We compared *A. urinae* infection in two different inbred mouse strains frequently used to study UTI, so that we could compare our results to observations made for more well-recognized uropathogens. We also compared the *in vivo* behavior of four different *A. urinae* strains isolated from patients with UTI, UUI, OAB, and a woman with no lower urinary tract symptoms.

## RESULTS

### Genomic comparison of *A. urinae* strains in relation to clinical outcomes

**Fig. 1.**
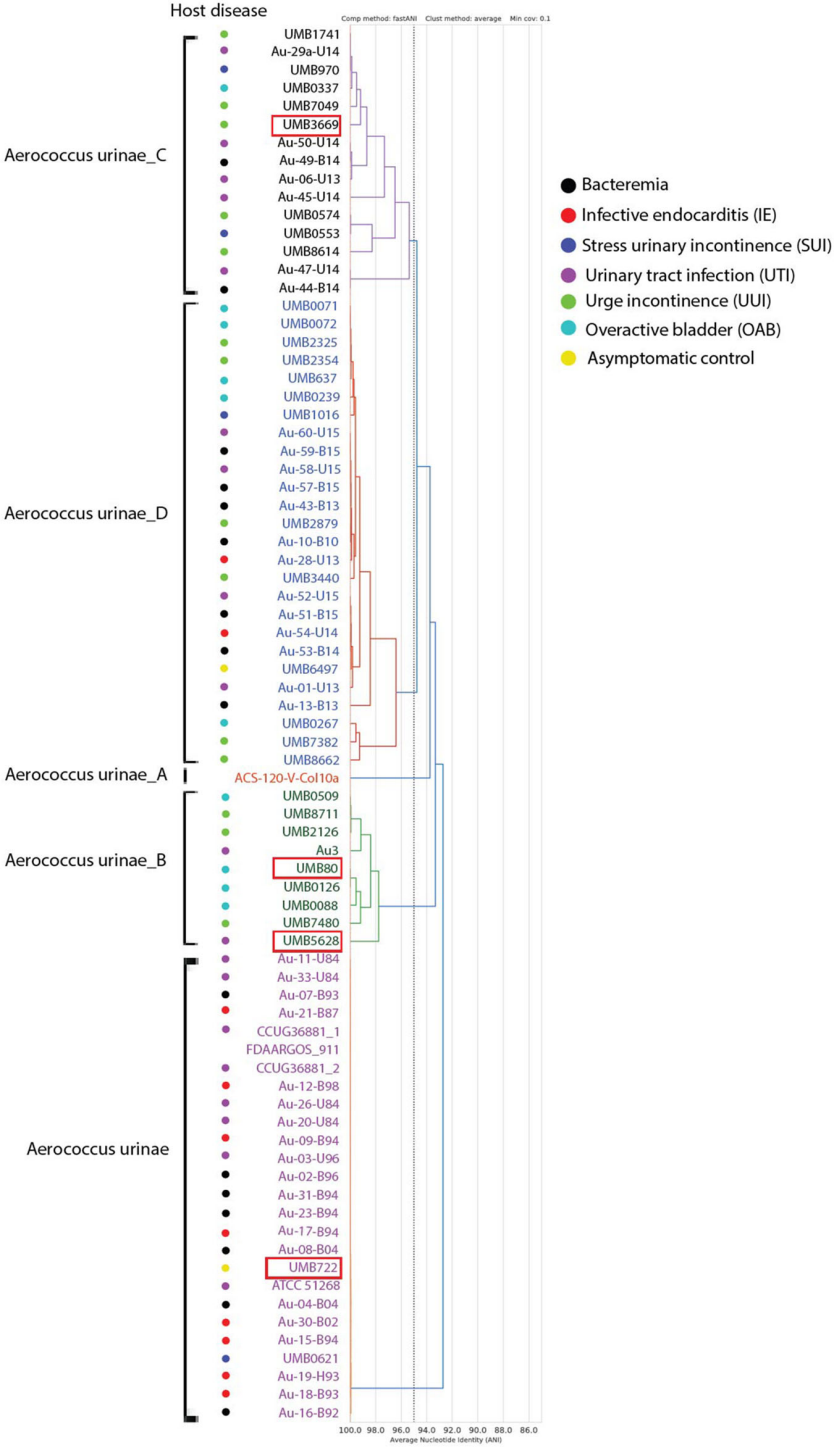
**Whole genome ANI analysis of clinical *A. urinae* strains*.*** The 77 clinical *A. urinae* strains cluster into five major groups based on a 95% ANI threshold. The dashed line indicates this threshold, and the five clades were assigned different colors. Host disease (UTI, UUI, SUI, OAB, bacteremia, and IE) of the clinical strains are marked with colored dots. Host disease information is unknown for strains FDAARGOS_911 and ACS-120-V-Col10A. Strains labelled UMB were isolated from urine collected by transurethral catheterization. Other strains were isolated from urine (U), blood (B) or heart valve (H). *Aerococcus urinae* clade names were chosen based on GTDB-tk calls. Red boxes denote strains used in mouse experiments.

To examine the potential relationship between phylogeny and infection outcomes of patients, we assembled the raw reads and compared the whole-genome average nucleotide identity (ANI) of the 77 publicly available *A. urinae* genomes (Fig. 1). These *A. urinae* strains comprised clinical isolates from cases of UTI, bacteremia, IE, OAB, UUI, and SUI, as well as two isolates from women with no urinary tract symptoms (asymptomatic controls). While these strains clustered into five distinct clades based on their ANI values, there was no apparent relationship between host disease group (UTI, UUI, SUI, OAB, bacteremia, and IE) and clade (ANOVA *P*=0.24 by clade; *P*=0.22 by symptom group). With the exception of the controls, each disease status was represented in at least three of the four clades that contained multiple isolates (B–E) (Table S1). This new genome comparison supports the conclusion that genome evolution alone is not a main determinant of clinical outcomes of *A. urinae* infection.

### Experimental *A. urinae* urinary tract infection depends on mouse genetic background

Using the data in Fig. 1, we chose four *A. urinae* strains from our collection to examine in the urinary tract of a mouse model. These strains were from three different clades and were isolated from patients with distinct clinical diagnoses: UMB80, isolated from a postmenopausal woman with OAB (clade: *Aerococcus urinae*_B); UMB3669, isolated from a postmenopausal woman with UUI (clade: *Aerococcus urinae*_C); UMB5628, isolated from a woman with a clinical UTI (clade: *Aerococcus urinae*_B), where *A. urinae* was the predominant uropathogen identified by culture; and UMB722, isolated from a control woman with no lower urinary tract symptoms or other apparent urological conditions (clade: *Aerococcus urinae*). These four strains have been characterized extensively *in vitro* in terms of aggregative properties (Hilt et al., 2020). When prepared in PBS as for mouse experiments, the strains appeared similar to each other as determined by transmission electron microscopy (TEM) analysis (Fig. S1).

We performed experiments in female C3H/HeN and C57BL/6 mouse strains, both of which have been used extensively in mouse models of UTI with Gram-positive and Gram-negative uropathogens. Of note, C3H/HeN mice have inherent vesicoureteral reflux (VUR), whereby urine travels in a retrograde fashion from the bladder up through the ureters and into the kidneys. In each experiment, the bladder was inoculated transurethrally with 0.5–1×10^7^ colony forming units (CFU) *A. urinae* in 50 μl PBS. We first examined the time course of *A. urinae* bacteriuria. In young C57BL/6 mice, all *A. urinae* strains were cleared from the urine by 24 h post infection (hpi; Fig. S2A,B, closed symbols). Rapid clearance by 24 hpi occurred even if the inoculum dose was increased to 0.5–1×10^8^ CFU (Fig. S2C). Since *A. urinae* often causes infection in older individuals and was frequently detected in postmenopausal women (Rasmussen, 2013; Price et al., 2020), we examined whether bacteriuria would better persist in older C57BL/6 retired breeders (Rb). While *A. urinae* titers 3 hpi were higher in Rb mice, nearly all mice cleared bacteriuria by 24 hpi (Fig. S2A,B, open symbols). These results demonstrate that wild-type female C57BL/6 mice do not represent an optimal host for modeling *A. urinae* urinary tract pathogenesis.

**Fig. 2.**
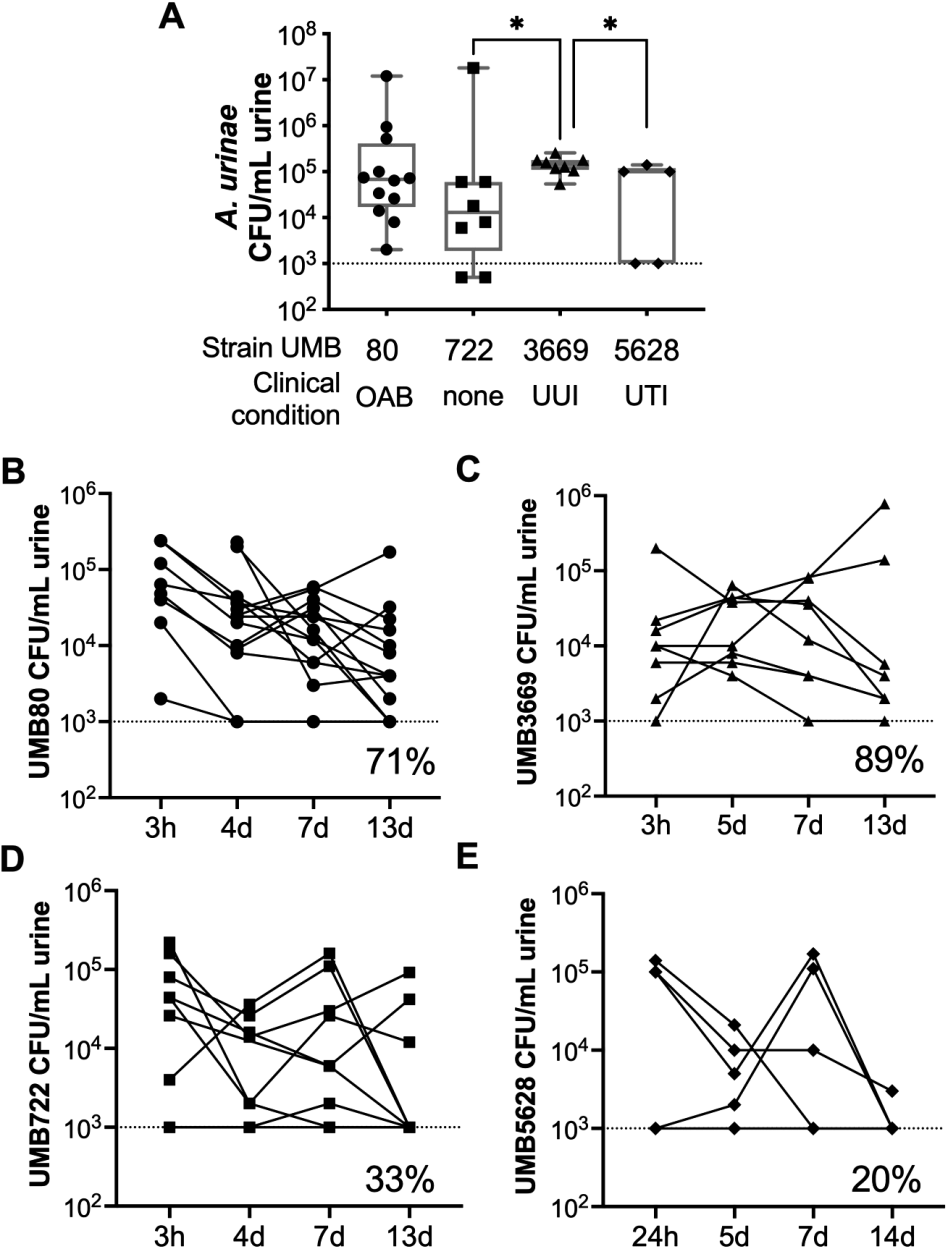
**Acute and persistent bacteriuria displayed by *A. urinae* strains.** (A) Titers of each *A. urinae* strain in urine collected 24 hpi. The clinical type of each isolate is indicated across the bottom of the graph. *P<0.05 Mann–Whitney test. (B–E) Time courses of bacteriuria caused by UMB80 (B), UMB3669 (C), UMB722 (D) and UMB5628 (E). Dotted lines on each graph denote the limit of detection (1000 CFU/ml). The percentage of mice with persistent bacteriuria out to 13 dpi is indicated on each graph.

In contrast to results in C57BL/6 mice, all *A. urinae* strains tested caused some level of acute bacteriuria 24 hpi in C3H/HeN mice (Fig. 2A). This result is consistent with previous reports with other Gram positive uropathogens (Kline et al., 2010, 2011). We wondered whether the persistence of *A. urinae* in C3H/HeN mice was simply a reflection of the presence of VUR in this mouse strain. However, the urogenital bacteria *Gardnerella vaginalis* was cleared from C3H/HeN mice by 12 hpi (Fig. S3). Therefore, persistent bacteriuria is not a non-specific phenotype that results from the inoculation of any urogenital bacterial species into the urinary tract of C3H/HeN mice. Coupled with their widespread use for studying other established uropathogens, these results support the utility of C3H/HeN mice to study *A. urinae* UTI.

### *Aerococcus urinae* strains cause different levels and durations of bacteriuria

There was variation among strains in the level of acute bacteriuria (Fig. 2A) and the rate of persistent bacteriuria out to 13 days post infection (dpi); 71% (10/14) for UMB80, 89% (8/9) for UMB3669, 33% (3/9) for UMB722, and 20% (1/5) for UMB5628 (Fig. 2B–E). It should be noted that, although the inoculum dose was the same for each strain as determined by CFU, the decreased ability of UMB722 to cause persistent infection is consistent with poorer growth in BHI compared to the other strains (Fig. S4). However, UMB5628 grew just as well, or better, than the other strains in BHI and nonetheless was cleared from most mice. Although UMB3669 was best able to cause persistent infection, this strain is hyper-aggregative *in vitro*, which could have resulted in more total bacteria being inoculated and also could confound CFU results in urine if similar aggregation occurs *in vivo*. Therefore, for further characterization in the C3H/HeN mouse model, we chose UMB80 because it caused persistent bacteriuria, consistently grows well, and is not aggregative in BHI media or when resuspended in PBS.

### *Aerococcus urinae* bacteriuria is dose-dependent

**Fig. 3.**
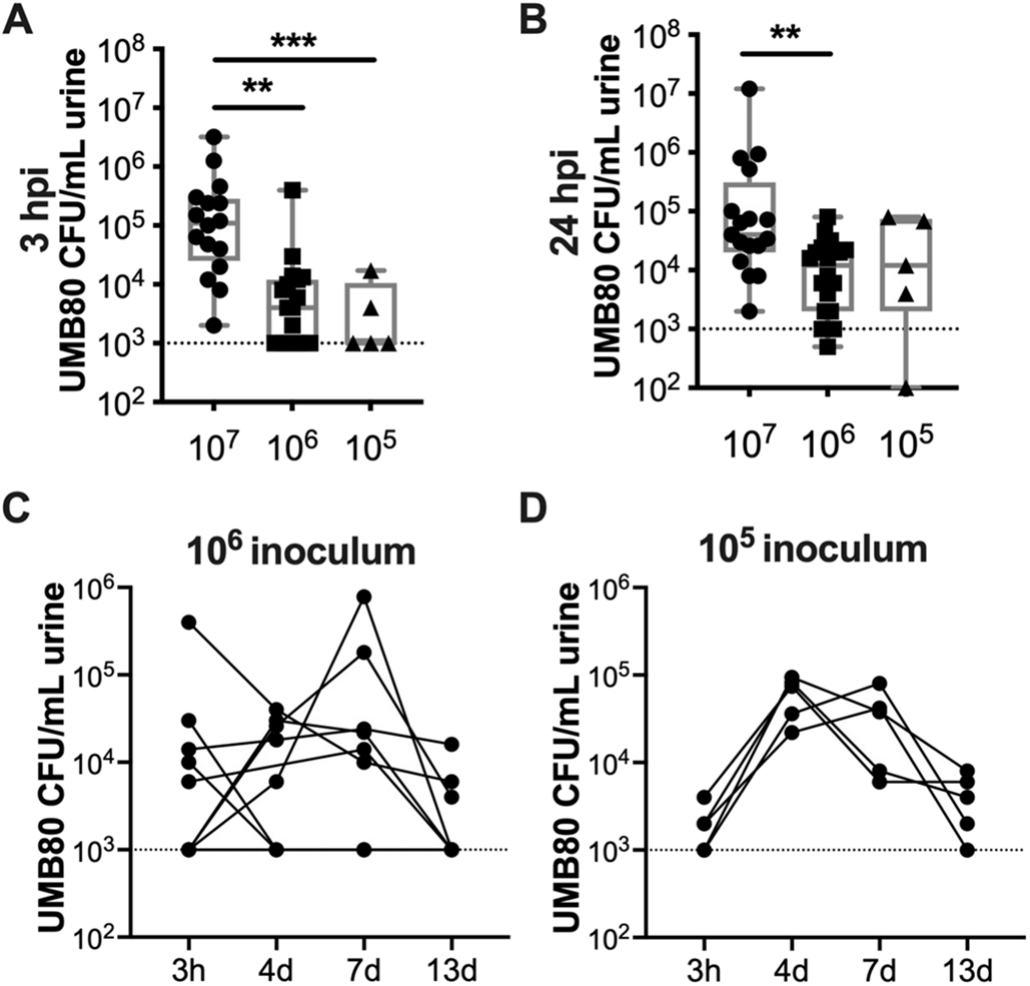
**UMB80 bacteriuria in mice depends on inoculum dose.** (A,B) Comparison of acute bacteriuria caused by different UMB80 inoculum doses in C3H/HeN mice at 3 hpi (A) and 24 hpi (B). Mann–Whitney test ***P<0.001, **P<0.01. (C,D) Time course of bacteriuria caused by the indicated doses of UMB80 in C3H/HeN mice. All results are combined from two independent experiments and each dot represents an individual mouse.

We performed additional experiments with UMB80 to determine the effect of modulating the inoculum dose on the time course of bacteriuria. Bacteriuria was dose-dependent, with inocula of 10^7^ CFU causing the highest levels of acute bacteriuria 3 and 24 hpi (Fig. 3A,B) that persisted for at least 13 dpi in the majority of mice (Fig. 2B). Mice inoculated with 10^6^ CFU of UMB80 displayed a variable pattern of bacteriuria (Fig. 3C). Mice inoculated with 10^5^ CFU of UMB80 had low levels of bacteriuria acutely (Fig. 3A,B), followed by proliferation between 3 hpi and 4 dpi, and then a steady decline out to 13 dpi (Fig. 3D).

### *Aerococcus urinae* does not colonize or cause exfoliation or inflammation in the bladders of C3H/HeN mice

**Fig. 4.**
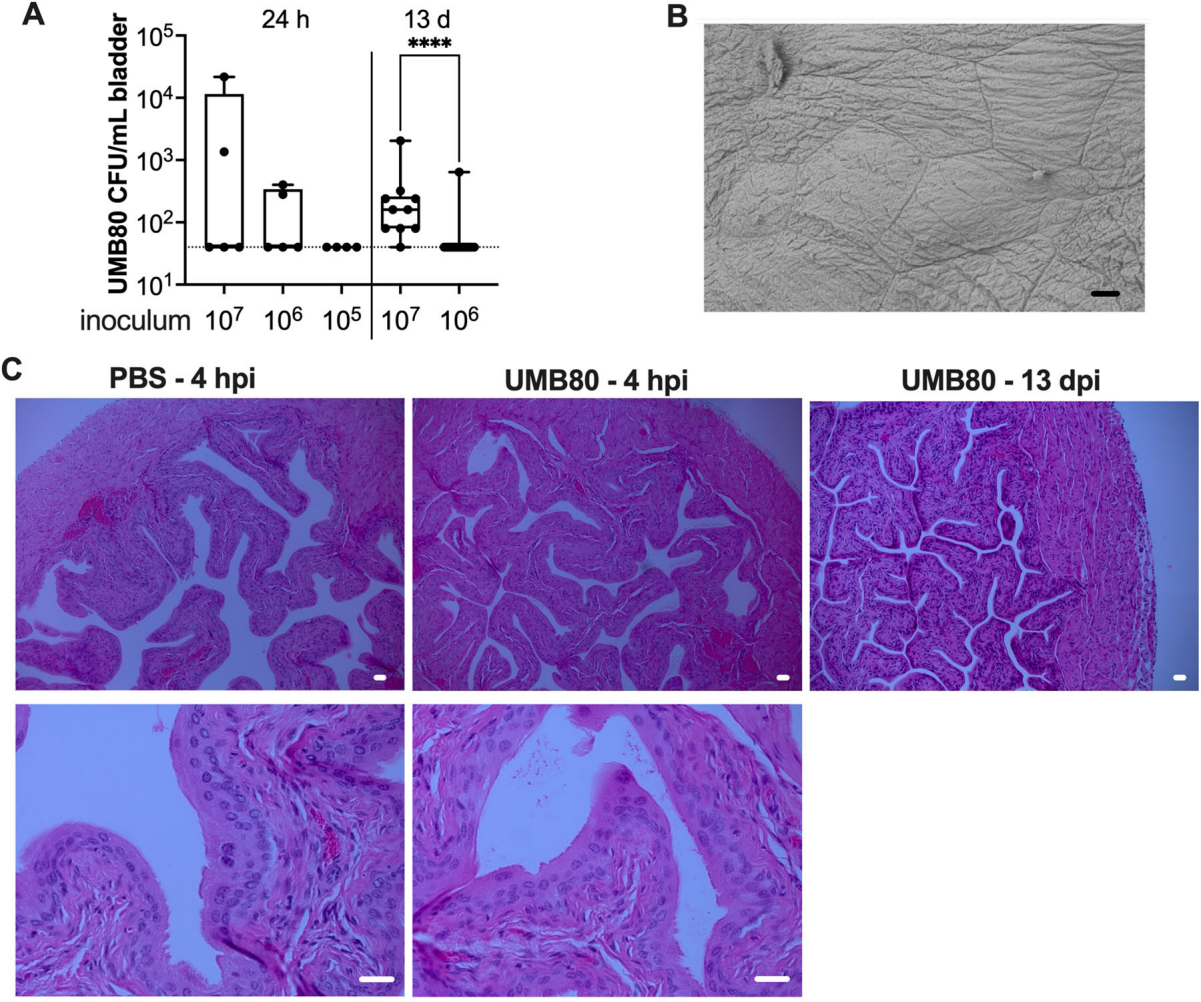
**UMB80 is rapidly cleared from the bladder without apparent uroepithelial exfoliation or histological inflammation.** (A) Titers of UMB80 in bladder tissue homogenates collected at the indicated time points. Each dot represents an individual mouse. (B) Scanning electron microscopy image of the luminal surface of a bladder from a mouse inoculated with UMB80, collected 6 hpi. Scale bar: 50 µm. (C) Formalin-fixed, paraffin-embedded bladder sections stained with Hematoxylin and Eosin (H&E). Scale bars: 50 µm.

Previous studies have shown that C3H/HeN mice with high levels of bacteriuria after inoculation with UPEC have similarly high bacterial loads in bladder and kidney tissues (Hannan et al., 2010). Thus, we were surprised to find that the levels of UMB80 were quite low in the bladders of mice 24 hpi, with many bladders harboring no detectable CFU (Fig. 4A). Though these data visually suggested a dose-dependent effect on bladder titers, there were no statistically significant differences based on inoculum dose at this time point. At 13 dpi, 10 of 14 (71%) mice inoculated with 10^7^ UMB80 had detectable bladder CFU, and titers were significantly higher compared to those inoculated with 10^6^ CFU, among which only one mouse had bacteria remaining in the bladder (Fig. 4A). Of note, when detected, bacterial loads of UMB80 in bladder tissue were substantially lower than what has been reported for UPEC (Hannan et al., 2010).

Clearance of UMB80 from the bladder suggested an inability of the organism to effectively adhere to the urothelium. Consistent with this, examination of bladders collected 3 and 6 hpi by scanning electron microscopy failed to detect adherent *A. urinae* bacteria on the urothelial surface (Fig. 4B). The urothelium appeared intact, with no signs of the exfoliation response initiated by other uropathogens (Anderson et al., 2003; O'Brien et al., 2017; Gilbert et al., 2017). Upon histological examination of formalin-fixed, paraffin-embedded sections stained with Hematoxylin and Eosin (H&E), the appearance of the transitional urothelium and superficial umbrella cells 4 hpi and 13 dpi was indistinguishable from that of PBS-inoculated control mice (Fig. 4C). We also did not observe the robust infiltration of neutrophils or edema in the bladder that are hallmark features of UPEC chronic cystitis (Hannan et al., 2010). Like UMB80, the additional *A. urinae* strains tested were not detectable in bladder tissue in most mice (data not shown), and bladder histology was indistinguishable from mice inoculated with PBS (Fig. S5). These results point to a urinary tract source other than the bladder for the persistent *A. urinae* bacteriuria observed in Fig. 2 and indicate that the bladder is not be the primary niche for *A. urinae* during UTI.

### *Aerococcus urinae* exhibits tropism for the kidney in C3H/HeN mice

**Fig. 5.**
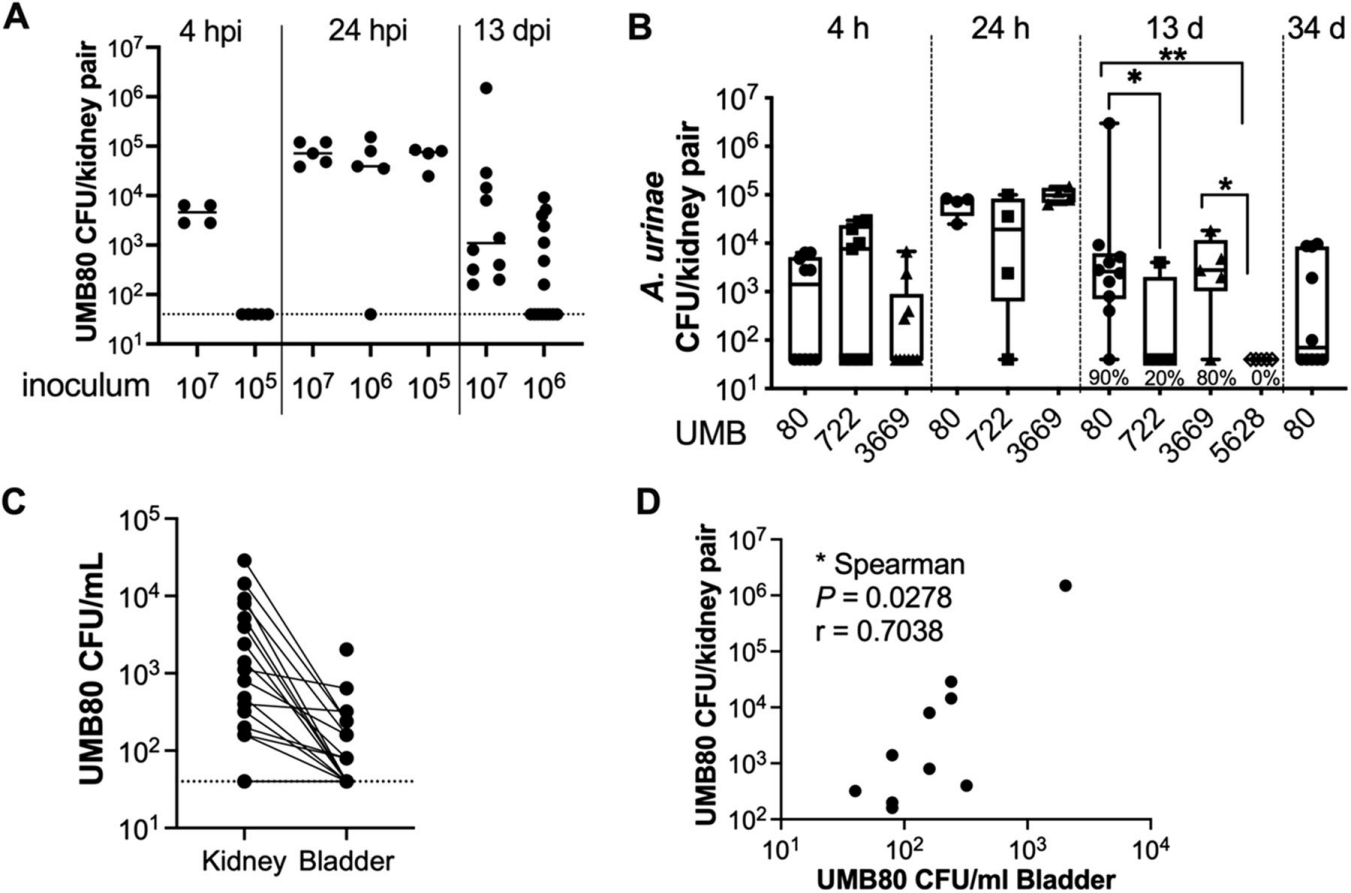
***Aerococcus urinae* causes acute and persistent kidney infection.** (A) Titers of UMB80 in kidney homogenates collected at the indicated time points following transurethral inoculation of the indicated doses in C3H/HeN females. (B) Titers of additional *A. urinae* strains in kidney homogenates collected at the indicated time points. *P<0.05, **P<0.01, Mann–Whitney test. Rate of persistent kidney infection indicated under the 13 dpi titers. (C) Correlation between kidney and bladder titers 13 dpi in mice receiving 10^7^ CFU UMB80. (D) Kidney titer data from 10^6^ CFU dose in panel A plotted relative to the bladder titer data shown in Fig. 4A, demonstrating that kidney infection occurred independent from bladder infection. In all graphs, each dot represents an individual mouse.

C3H/HeN mice exhibit vesicoureteral reflux, enabling bacteria inoculated into the bladder to rapidly reach the kidney. Titers of UMB80 were detected in the kidneys 4 hpi with an inoculum of 10^7^ CFU, but not with 10^5^ CFU. However, by 24 hpi, UMB80 was detected at high levels in the kidneys, irrespective of inoculum dose (Fig. 5A). This is consistent with the urine titer results that indicate expansion of UMB80 in mice receiving a lower inoculum dose (Fig. 2D). Kidney infection at early timepoints following inoculation was a shared feature among the additional *A. urinae* isolates tested (Fig. 5B). However, differences were observed between strains at later time points. At 13 dpi, the presence of detectable titers in kidneys reflected the persistent bacteriuria results shown in Fig. 2; UMB3669 exhibited similar levels of kidney infection as UMB80, whereas UMB722 and UMB5628 were not detected in most kidney homogenates at this time point (Fig. 5B). The rate of persistent kidney infection out to 13 dpi for each *A. urinae* strain was: 90% (9/10) for UMB80, 20% (1/5) for UMB722, 80% (4/5) for UMB3669 and 0% (0/5) for UMB5628. In an additional experiment, four of ten mice inoculated with 10^7^ UMB80 had detectable titers in kidney tissue 34 dpi (Fig. 5B). Of particular note, over half (7/12) of the mice that had detectable UMB80 titers in kidney tissue did not have detectable bladder titers (Fig. 5C). In mice with detectable UMB80 in both tissues, the titers in bladders were significantly correlated with bacterial loads in the kidneys (Fig. 5D). This result is consistent with the conclusion that *A. urinae* detected in bladders at 13 dpi reflects bacteria descending from the infected kidney. Together, these results demonstrate that some strains of *A. urinae* can cause persistent kidney infection in C3H/HeN mice independent of infection in the bladder.

**Fig. 6.**
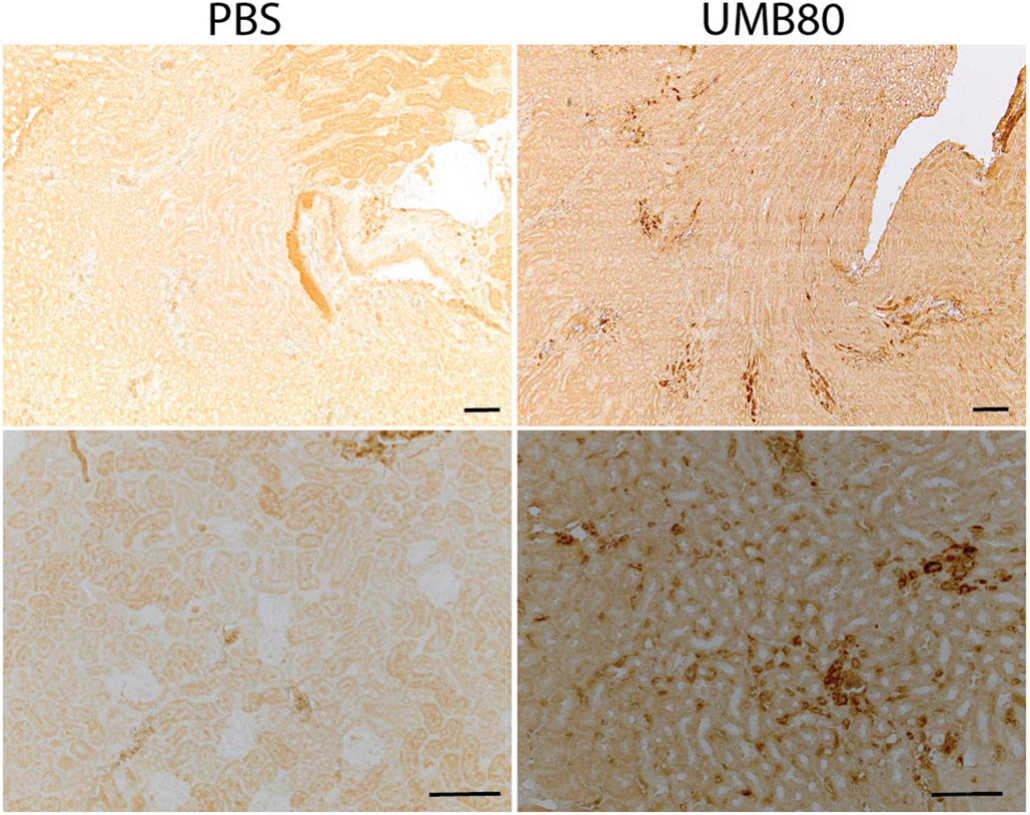
**Localization of *A. urinae* in kidney tissue kidney.** Formalin-fixed, paraffin-embedded kidney sections collected 13 dpi from mice inoculated with either PBS or UMB80 and stained with primary antibody to the Gram-positive cell wall component peptidoglycan. Scale bars: 100 µm.

To determine the spatial localization of UMB80 within the kidney, we performed immunohistochemistry (IHC) with an antibody to peptidoglycan, a component of the cell wall of Gram-positive bacteria. This analysis revealed stained regions presumably representing collections of *A. urinae* bacteria (Fig. 6; Fig. S6). Positive staining was observed emanating from the collecting system into the medulla and was in some mice detected in the cortex. The signal was not contained within tubules, where UPEC has been shown to localize during kidney infection (Olson et al., 2018), but instead appeared to be in inter-tubular spaces. Since the primary antibody detects a cell wall component, it is possible that some of the staining detected free peptidoglycan released from the bacteria. Importantly, positive signal was not present in kidney sections collected from mice inoculated with PBS and stained in parallel.

### *Aerococcus urinae* causes histological inflammation and neutrophil recruitment to the kidney

**Fig. 7.**
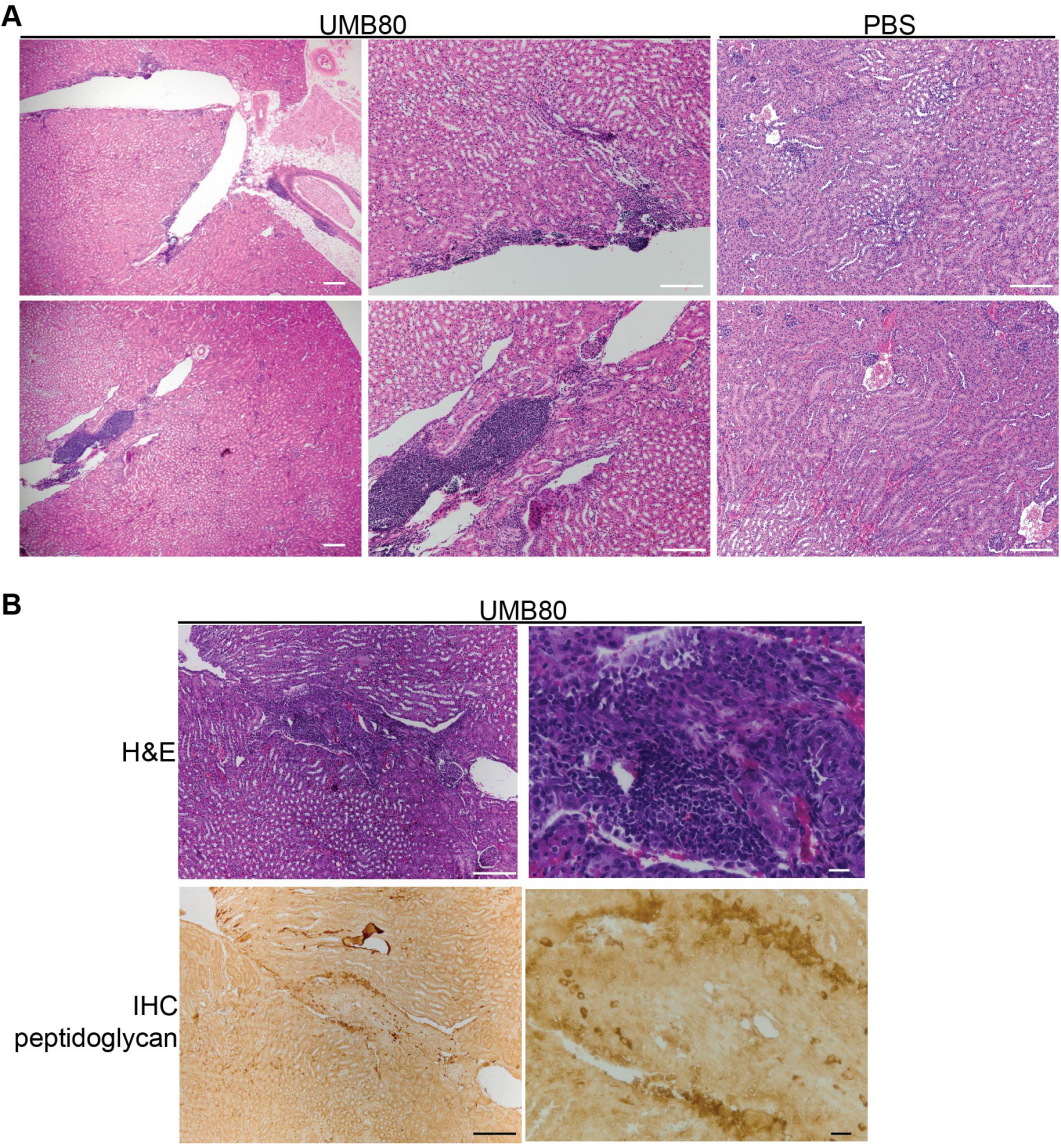
**UMB80 triggers histological inflammation in the kidney.** (A) Formalin-fixed, paraffin-embedded kidneys collected 13 dpi from mice transurethrally inoculated with PBS or UMB80 were sectioned and stained with H&E. Scale bars: 200 µm. (B) Adjacent kidney sections from a mouse inoculated with UMB80 stained with H&E or with an antibody to peptidoglycan. Scale bars: 20 µm.

**Fig. 8.**
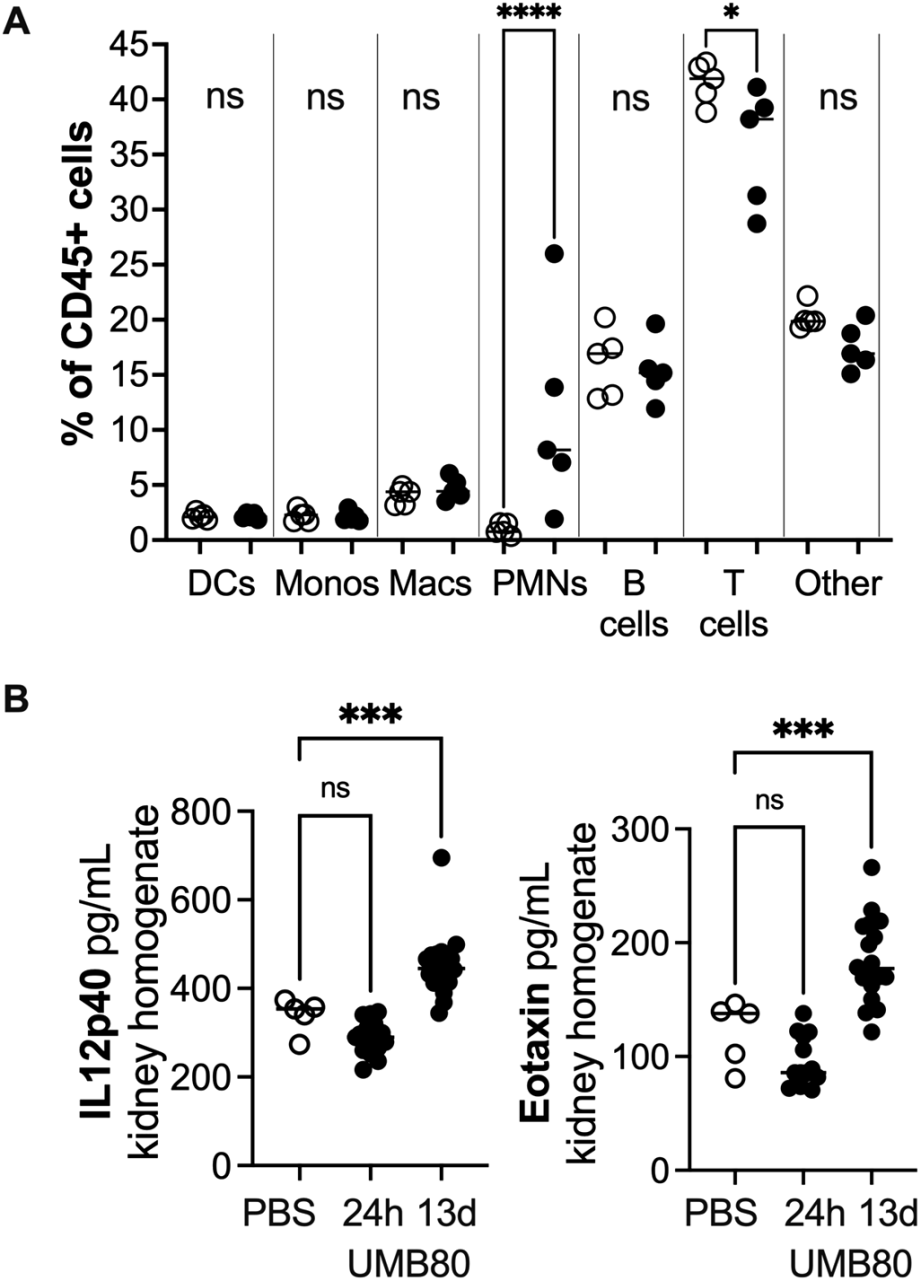
**Neutrophils (PMNs) and pro-inflammatory cytokines are increased by UMB80 in the kidney.** (A) Flow cytometry of immune cells in kidneys from mice inoculated with PBS (open symbols) or UMB80 (closed symbols), collected 13 dpi. One-way ANOVA followed by Šídák's multiple comparisons tests; ****P<0.0001; *P<0.05. (B) Cytokine levels in kidney homogenate supernatants collected at the indicated time points. One-way ANOVA followed by Dunnett's multiple comparisons tests; ***P<0.001; n.s., not significant.

In contrast to the bladders, the kidneys of mice inoculated with UMB80 displayed histological inflammation 13 dpi (Fig. 7). The degree of inflammation ranged from smaller collections to large patches of inflammatory cells (Fig. 7A). Of note, some regions of inflammation also showed positive staining with the anti-peptidoglycan antibody, suggesting that the inflammation was directed as sites of active infection (Fig. 7B). To determine the identity of the immune cells infiltrating or expanding in the kidney, we performed flow cytometry profiling of kidney tissue. This analysis revealed a significant increase in neutrophils in the kidneys of UMB80-infected mice compared to mock-infected controls (Fig. 8A). We also profiled the levels of 23 cytokines in kidney tissues at 24 hpi and 13 dpi (Fig. S7). Kidneys from UMB80 infected mice had significantly higher levels of IL-12p40 and Eotaxin compared to control animals (Fig. 8B). While the source of these cytokines remains to be determined, in the context of other microbial infections, neutrophils have been shown to either produce 1L-12p40 themselves or induce production by other immune cells such as DCs (Bliss et al., 2000; Bennouna et al., 2003). Eotaxin is primarily produced by epithelial cells and endothelial cells. Previous data showed that C3H/HeN mice with chronic UPEC bacteriuria and kidney infection have abundant neutrophils in urine (Hannan et al., 2010). However, we did not observe neutrophils upon cytological analysis of urines collected 13 dpi from mice infected with UMB80 or the other *A. urinae* isolates tested (data not shown). Together, these data demonstrate that UMB80 preferentially infects the kidney in C3H/HeN mice and triggers a neutrophilic inflammatory response that remains contained within the tissue.

## DISCUSSION

*Aerococcus urinae* is an emerging uropathogen that can cause disseminated and potentially life-threatening infections, especially in patients with predisposing risk factors such as urinary tract abnormalities. While the pathogenic potential of different *A. urinae* strains has been examined using genomic and *in vitro* phenotypic data, infection outcomes have not previously been examined *in vivo*. Here, we present a mouse model of *A. urinae* infection that enables examination of tissue tropism and host immune responses. *A. urinae* displayed a different course of infection in two genetically and phenotypically distinct mouse strains. Consistent with *A. urinae* infections being predominantly reported in patients with underlying risk factors, including urinary tract abnormalities, *A. urinae* caused more robust infection in mice with an inherent urinary tract abnormality (VUR). As with any small animal model system, the C3H/HeN mouse model has limitations, and it cannot be used to understand all instances of *A. urine* UTI observed in humans. For example, the absence of persistent bladder infection in mice by the *A. urinae* limits the ability to study *A. urinae-*urothelial interactions *in vivo*. Examination of additional *A. urinae* strains could identify some more capable of infecting the bladder. It is important to note here that it is not yet known whether *A. urinae* binds to the human bladder urothelium during UTI. In addition to the expanded studies mentioned below, the present model could be adapted to examine *A. urinae* infection outcomes in the context of additional predisposing factors, such as by adapting the established catheter-associated UTI (CAUTI) mouse model (Guiton et al., 2010) in female mice. Additionally, the model could be adapted to examine sex differences in infection using established models of UTI in male mice (Olson et al., 2016; Zychlinsky Scharff et al., 2017).

When comparing the results of this new *A. urinae* UTI mouse model to the well-established uropathogenic *E. coli* (UPEC) UTI model, there are some notable similarities and differences. Both UPEC and *A. urinae* are more rapidly cleared and kidney infection is rare in C57BL/6 compared to C3H/HeN mice (Hopkins et al., 1998). C3H/HeN mice are more susceptible to prolonged (chronic) bacteriuria and kidney infection because they have VUR, which facilitates bacterial ascension from the bladder to the kidney (Bowen et al., 2013). While persistent infection by both *A. urinae* and UPEC occurs in C3H/HeN mice, there is a distinction with respect to tissue tropism. C3H/HeN mice with chronic UPEC bacteriuria have high bacterial burden both in bladder and kidney tissue (Hannan et al., 2010), while *A. urinae* was undetectable in the bladder tissue of many mice with kidney infection. Also in contrast to chronic UPEC infection, mice with *A. urinae* infection and inflammation in kidney tissue did not have pyuria and displayed no signs of distress (lethargy, ruffled fur, hunched posture), suggesting that *A. urinae* may be capable of causing a persistent but localized, kidney infection. While it would be difficult to prove that *A. urinae* kidney colonization in the absence of typical pyelonephritis symptoms also occurs in humans, the possibility is consistent with the high frequency at which *A. urinae* disseminates from the urinary tract into the bloodstream to cause infection in other organs and tissues. Initial mouse experiments included culturing of spleens and did not detect disseminated *A. urinae*. Future studies could examine whether dissemination in mice occurs given a longer timeframe, or higher inoculum dose, or in immunocompromised mice.

Notable comparisons to other Gram-positive UTI models can be made as well. Similar to *A. urinae*, transurethral inoculation of *Staphylococcus saprophyticus* into C3H/HeN female mice demonstrated higher bacterial titers in the kidney compared to the bladder. Persistence of bacteria was noted in C3H/HeN mice but not in C57BL/6 mice (Kline et al., 2010). Infection of Group B *Streptococcus* (GBS) and *Enterococcus faecalis* under similar conditions also demonstrated a kidney tropism (Kline et al., 2011; Kau et al., 2005). This preference and persistence pattern of Gram-positive uropathogens towards the kidneys over the bladder lends itself to further investigation of UTI pathogenesis.

Outside of the context of a clinical UTI, two studies found *A. urinae* more frequently present as part of the ‘urinary microbiome’ in women with UUI (Pearce et al., 2014; Price et al., 2020). Of note, women with UUI in these studies were older and more were estrogen negative than women in the control group. Whether or not *A. urinae* colonization is a cause or consequence of UUI, or is dependent upon age or hormone status, remains to be determined. The results presented here from bladder titers and microscopy do not provide any evidence for *A. urinae* binding to the bladder urothelium in mice. This is perhaps not surprising because *A. urinae* UTI would be expected to be more common if it were capable of binding and invading superficial urothelial cells in a similar fashion as professional uropathogens like UPEC (Terlizzi et al., 2017). The observation that *A. urinae* was rapidly cleared from the bladder in mice suggests that some perturbation of the urothelium may be required to facilitate *A. urinae* in the bladder. It is possible that women with UUI have an altered urothelium that facilitates *A. urinae* bladder colonization, but so far bladder tissues from UUI patients have not been examined. Alternatively, the presence of *A. urinae* in the urine of women with UUI could reflect infection of the kidney. It is reasonable to speculate that if *A. urinae* kidney infection and inflammation (without overt signs of pyelonephritis) similar to what we observed in mice also occurs in humans, it could result in lower urinary tract symptoms such as those associated with UUI. Future studies could examine *A. urinae* infected mice for the presence of incontinence, examine cytokines and chemokines in urine, and could determine whether *A. urinae* bladder colonization is enhanced in established mouse models of urinary incontinence (Jiang and Damaser, 2011; Parsons and Drake, 2011).

Our ANI analysis is consistent with a previous study that did not find a relationship between *A. urinae* phylogeny and disease status (Carkaci et al., 2017). The 77 available *A. urinae* genomes separated into five distinct clades, but none had an overrepresentation of isolates from a particular disease state (e.g. UTI or bacteremia). The lack of association with disease status suggests that all five clades contain pathogenic strains. Since an ANI value of 95% is typically considered the boundary for bacterial species (Jain et al., 2018), our result suggests that *A. urinae* may consist of five closely related species. Efforts are underway to test this hypothesis. Likewise, while we observed differences in infection outcomes between *A. urinae* isolates, these differences did not track with the type of infection or lower urinary tract condition from which the strain was isolated. We acknowledge the limitation that we examined only a single strain from each clinical outcome and did not examine a bacteremia isolate. Future studies with additional *A. urinae* isolates are needed to establish whether UTI or bacteremia isolates in general are more pathogenic. Taken together, our results suggest that host factors, rather than bacterial ones, are a main driver of *A. urinae* infections and are consistent with the concept that *A. urinae* is primarily an opportunistic pathogen.

Previous studies have examined *in vitro* phenotypes that are potentially important for pathogenesis. An analysis of five *A. urinae* strains isolated from IE cases reported that plasma components could stimulate platelet aggregation and biofilm formation (Shannon et al., 2010). We recently reported self-aggregative properties of 24 *A. urinae* strains isolated primarily from postmenopausal women with UUI or OAB (Hilt et al., 2020). A survey of the surface proteome of *A. urinae* strains led to the characterization of two cell wall-anchored proteins that are being studied for their adhesive properties (Senneby et al., 2019). Future studies could examine the role of these, or other phenotypes, for pathogenesis by comparing *A. urinae* strains in the mouse model. Such efforts would be greatly enhanced by the development of a genome-editing platform in *A. urinae*, similar to those that have been successful with other Gram-positive uropathogens (Keller et al., 2019; Penewit and Salipante, 2020).

We describe in this report a mouse model that offers to serve for the study of bacterial and host factors in the pathogenesis of *A. urinae* in the urinary tract. It will be especially useful for studying *A. urinae* in the kidney, although future studies may also reveal *A. urinae* strains capable of causing more persistent bladder infection. Given how little is known about the mechanisms and behaviors of infection, this model should aid in understanding the role of *A. urinae* in urinary tract diseases.

## MATERIALS AND METHODS

### Genome analysis: read quality control, contigs assembly and genome quality assessment

All publicly available raw reads for *A. urinae* whole genome sequencing projects were downloaded from SRA (https://www.ncbi.nlm.nih.gov/genome/browse/#!/prokaryotes/3029/) and ENA (https://www.ebi.ac.uk/ena/browser/view/PRJEB36767). The quality of the raw reads was assessed using FastQC (https://www.bioinformatics.babraham.ac.uk/projects/fastqc/) and trimmed using bbduk (https://jgi.doe.gov/data-and-tools/bbtools/). Bases with low quality scores (qtrim=rl, trimq=10) and positions with high compositional bias (ftl=15, ftr=135 or 239 depending on the samples) were removed from both ends. Only reads with minimum read lengths of 30 bp (minlength=30), with no Ns (maxns=0), and with an average quality above 10 (maq=10) were kept. After quality control, all of the clean paired-end reads were assembled using SPAdes (v3.14.1) (Bankevich et al., 2012) with the (--isolate) mode. The full k-mer size list (-k 21, 33, 55, 77, 99, 127) or (-k 21,33,55,77) was used in the assembly depending on the sequence length of samples. CheckM (v1.0.12) (Parks et al., 2015) was then used to assess completeness and contamination of the assembled genomes using the ‘lineage_wf’ pipeline, and genomes were filtered at completeness ≥90% and contamination ≤5% to obtain high-quality genomes. Samples with contamination above 5% were then filtered using MaxBin 2.0 (v2.2.7) (Wu et al., 2016) to recover high-quality genome bins. dRep (v2.2.3) (Olm et al., 2017) was used to compare the average nucleotide identity (ANI). 95% ANI values calculated by fastANI (Jain et al., 2018) were used to cluster genomes into different groups (-sa 0.95 --S_algorithm fastANI --SkipMash). An ANOVA was performed considering ANI values by clade and symptom group using R (v4.0.5).

### Ethics statement

Mouse experiments were carried out in strict accordance with the recommendations in the Guide for the Care and Use of Laboratory Animals. The Institutional Animal Care and Use Committee (IACUC) of Washington University School of Medicine approved the protocols (Protocol Number: 20170081 and 20-0031).

### Mice

Six- to seven-week-old female C57BL/6 mice were obtained from Charles River (Fredericks facility), and C3H/HeN mice were obtained from Envigo International Holdings. Mice were given a regular chow diet in a specific pathogen-free facility at Washington University School of Medicine.

### Bacterial strains and growth conditions

*Aerococcus urinae* strains UMB80, UMB722, and UMB3669 were compared side-by-side in three independent mouse experiments and an additional experiment compared UMB80 and UMB5627. The strains were first streaked from frozen stocks onto BHI agar plates and grown in 5% CO_2_ atmospheric conditions at 37°C for 48 h. These plates were used to start 10 ml liquid BHI cultures incubated statically under the same conditions overnight (∼18 h). Prior to performing mouse experiments, we empirically determined the appropriate OD_600_ equivalent required to achieve an ∼10^7^ CFU (as determined by serial dilution plating) dose in 50 µl PBS for each strain. We found that UMB80 needed to be concentrated to an OD_600_ of 10 and UMB722 needed to be concentrated to an OD_600_ of 5. Because of the aggregation of UMB3669, we did not rely on OD determination, but found that we could achieve the desired inoculum dose when resuspending the culture pellet in the same volume of PBS that was determined for UMB722. For mouse experiments, the overnight cultures were centrifuged and the bacterial pellets were re-suspended in sterile PBS. The inocula were serially diluted and plated on BHI media to confirm that the target CFU dose was achieved. Of note, trituration of UMB3669 in PBS facilitated dispersal of large aggregates and achieved a homogenous and suspended inoculum.

A streptomycin-resistant strain of *Gardnerella vaginalis* JCP8151B was streaked from frozen stock on NYCIII agar plates and grown at 37°C for ∼24 h in a Coy anaerobic chamber. These plates were used to start 3 ml NYCIII static liquid anaerobic cultures incubated at 37°C overnight for ∼18 h. *G. vaginalis* inoculum was prepared in PBS at OD 5 (∼5×10^7^–1×10^8^ CFU in 50 μl).

### Mouse urinary tract inoculation experiments

Mice were anesthetized with isoflurane and then inoculated transurethrally with 50 μl prepared inocula as previously described (Kline et al., 2012, 2014). Urine was collected from mice and monitored for *A. urinae* titers by dilution plating on BHI supplemented with 50 μg/ml kanamycin. This selective plating strategy takes advantage of the natural kanamycin resistance of these four *A. urinae* strains (not shown) and was necessary because ‘clean catch’ urine is not possible from a mouse; resident urogenital microbiota inevitably grow on non-selective BHI media and preclude enumeration of *A. urinae*. At the experimental endpoint, mice were humanely sacrificed (cervical dislocation under anesthesia), and bladders and kidneys were aseptically harvested, kept on ice, and homogenized in PBS for serial dilution and plating to enumerate *A. urinae* titers on BHI+kan media. Initial experiments also collected spleens, but disseminated *A. urinae* were not detected. Remaining homogenates were centrifuged 13,000 rpm in a benchtop microcentrifuge for 5 min at 4°C to remove debris and supernatants were stored at −20°C for cytokine analysis.

### Growth curves

Growth curves of *A. urinae* strains were measured by optical density at 600 nm with an Epoch 2 microplate reader (BioTek Instruments Inc.). The strains were first streaked from frozen stocks onto BHI agar plates and grown in 5% CO_2_ atmospheric conditions at 37°C for 48 h. Strains were then inoculated into 5 ml liquid BHI medium in a six-well plate (Corning, Inc.) and read with shaking at 200 rpm for 48 h kept at 5% CO­_2_ atmospheric conditions. An additional read was conducted with the same strains passaged 1:10 into fresh media for another 48 h.

### Transmission electron microscopy

For ultrastructural analyses, *A. urinae* cells prepared in PBS (as for mouse inoculations) were spun at 500 rpm; pellets were fixed in 2% paraformaldehyde/2.5% glutaraldehyde (Polysciences Inc., Warrington, PA, USA) in 100 mM sodium cacodylate buffer, pH 7.2 for 1 h at room temperature. Samples were washed in sodium cacodylate buffer at room temperature and postfixed in 1% osmium tetroxide (Polysciences Inc.) for 1 h. Samples were then rinsed extensively in dH_2_O prior to en bloc staining with 1% aqueous uranyl acetate (Ted Pella Inc., Redding, CA, USA) for 1 h. Following several rinses in dH_2_O, samples were dehydrated in a graded series of ethanol and embedded in Eponate 12 resin (Ted Pella Inc.). Sections of 95 nm were cut with a Leica Ultracut UCT ultramicrotome (Leica Microsystems Inc., Bannockburn, IL, USA), stained with uranyl acetate and lead citrate, and viewed on a JEOL 1200 EX transmission electron microscope (JEOL USA Inc., Peabody, MA, USA) equipped with an AMT 8-megapixel digital camera and AMT Image Capture Engine V602 software (Advanced Microscopy Techniques, Woburn, MA, USA).

### Scanning electron microscopy

Bladders were fixed *in situ* as previously described (O'Brien et al., 2020) with EM fixative (2% paraformaldehyde, 2% glutaraldehyde in 0.1 M sodium phosphate buffer, pH 7.4). Samples were prepared by critical point drying. Briefly, samples were post-fixed in 1.0% osmium tetroxide, dehydrated in increasing concentrations of ethanol, and then dehydrated at 31.1°C and 1072 PSI for 16 min in a critical point dryer. Samples were mounted on carbon tape-coated stubs and sputter-coated with gold/palladium under argon. Bladders were imaged on a Zeiss Crossbeam 540 FIB-SEM.

### IHC analysis

Formalin-fixed, paraffin embedded bladders and kidneys were sectioned and stained with H&E by the histology core in the Department of Obstetrics and Gynecology at Washington University School of Medicine (St. Louis, MO, USA) or by Nationwide Histology (Missoula, MT, USA). For IHC, slides were deparaffinized in xylene followed by a graded series of ethanol washes. Slides were rehydrated in running water and antigen retrieval was performed by microwaving for 20 min in sodium citrate buffer, pH 6 with 0.05% Tween 20. Slides were blocked for 1 h with 1% BSA+0.3% Triton X-100 in PBS, probed for 1 h with a 1:150 dilution of monoclonal anti-peptidoglycan antibody (Invitrogen #MA5-16509) in same buffer, washed with PBS, and probed for 30 min with 1:200 dilution of biotinylated secondary antibody. Signal was detected using Vectastain Elite ABC reagent and DAB solution.

### Urine cytology

Eighty microliters of a tenfold dilution of urine was cytospun onto coated CytoPro Dual microscope slides and stained with a Hema 3 staining kit (Thermo Fisher Scientific) to visualize epithelial cells and neutrophils (PMNs). Slides were observed on an Olympus Vanox AHBT3 microscope

### Flow cytometry

Kidneys were harvested after terminal perfusion with PBS into cold RPMI. Manual dissociation was used to create a single-cell suspension. Cells were then treated with RBC lysis buffer (155 mM NH_4_Cl, 10 mM KHCO_3_) at room temperature to remove any remaining red blood cells. After washing, cells were subjected to a Percoll gradient (Percoll Plus; GE Healthcare, Uppsala, Sweden) in FACS buffer [10% FBS, 1% w/v sodium azide, 2 mM ethylenediaminetetraacetic acid (EDTA) in PBS]+25 mM sucrose for leukocyte enrichment, then resuspended in cold PBS and stained with Live/Dead Fixable Blue (Thermo Fisher Scientific). Following another wash, cells were blocked with CD16/CD32 antibody (BD Biosciences, San Jose, CA, USA) on ice and stained with fluorescently conjugated antibodies against the following extracellular antigens: MHC-II-BUV395 (BD Biosciences #BDB743876), CD115-BV480 (BD Biosciences #BDB746456), CD45-BV510 (BD Biosciences #563891), CD11b-BV570 (BioLegend #101233), Ly6C-BV605, CD19-Qdot655 (Invitrogen #501138153), CD11c-BV785 (BioLegend #117336), CD3-SparkBlue550 (BioLegend #100260), F4/80-BB700 (BD Biosciences #BDB746070), TCRγδ-PE-Cy5 (BioLegend), CD4-APC (BD Biosciences #553051), Ly6G-AlexaFluor 700 (BioLegend #127621), and CD8a APC-Cy7 (BD Biosciences #557654). Cells were washed, resuspended in FACS buffer and analyzed on an Aurora flow cytometer (Cytek Biosciences). Analysis was performed using the FlowJo software (BD Biosciences). Gating strategy used to determine immune cell types is illustrated in Fig. S8.

### Bioplex cytokine analysis

Tissue homogenate supernatants collected as described above were thawed on ice, centrifuged again at 4°C to remove any remaining particulates. Cytokine content was measured using the Bio-Plex-Pro Mouse Cytokine 23-Plex Panel multiplex cytokine bead kit (Bio-Rad), which quantifies 23 different cytokines and chemokines. The assay was performed according to manufacturer instructions, except using tenfold less standard and half the amount of coupled beads and detection antibodies indicated in the protocol.

### Statistics

The figures show individual data points for each animal with a line at the geometric mean or with box and whiskers (min. to max.) plots. The statistical tests used to analyze each set of data, as indicated in the figure legends, were determined using Prism 9 (GraphPad) software. For analysis of cytokines, raw uncorrected *P*-values are provided.

## Supplementary Material

Supplementary information
