# Supplementary material for: A mouse model displays host and bacterial strain differences in Aerococcus urinae urinary tract infection
Source: Biol Open. 2021 Aug 13;10(8):bio058931. doi: 10.1242/bio.058931 (PMC8380466; doi:10.1242/bio.058931)
Supplement: Supplementary information [file biolopen-10-058931-s1.pdf]

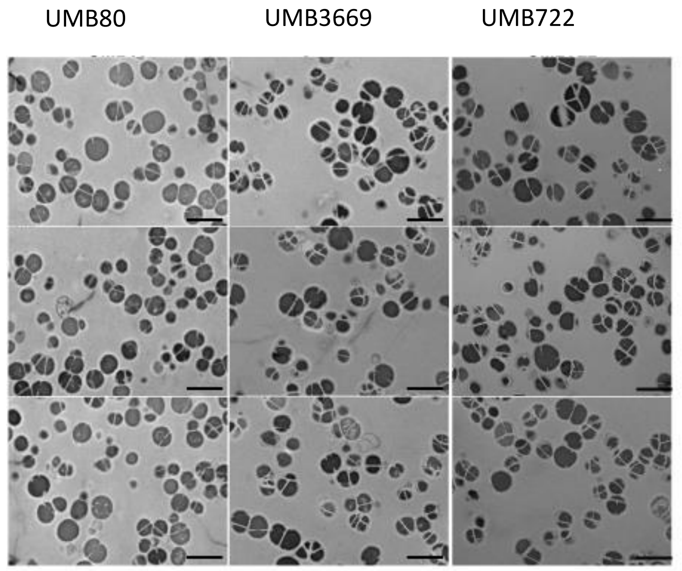

Fig. S1. Transmission electron microscopy of *A. urinae* strains used in mouse experiments.

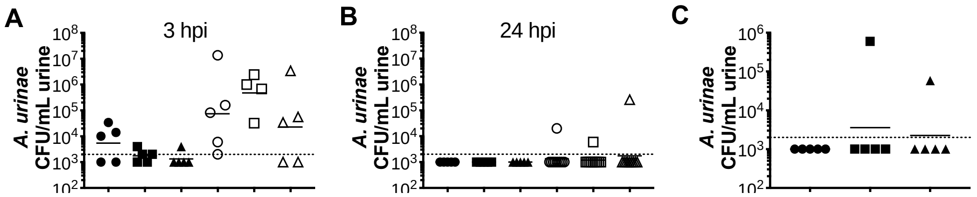

Fig. S2. *A. urinae* is rapidly cleared from female C57BL/6 mice. Titers of each *A. urinae* strain in urine collected at 3 hpi (A) or 24 hpi (B-C). Each dot represents an individual mouse. Closed symbols are data from young mice and open symbols are from retired breeders. Results are from two independent experiments with inoculum doses of 0.5-1 x 10^7 (A-B) or 0.5-1 x 10^8 CFU. Dotted lines on each graph denote the limit of detection (1000 CFU/mL).

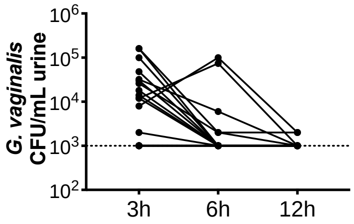

Fig. S3. ***Gardnerella vaginalis*** **is rapidly cleared from C3H/HeN mice. Titers of** ***G. vaginalis*** **strain 8151B collected at the indicated timepoints.** Each dot represents an individual mouse. Dotted line denotes the limit of detection (1000 CFU/mL).

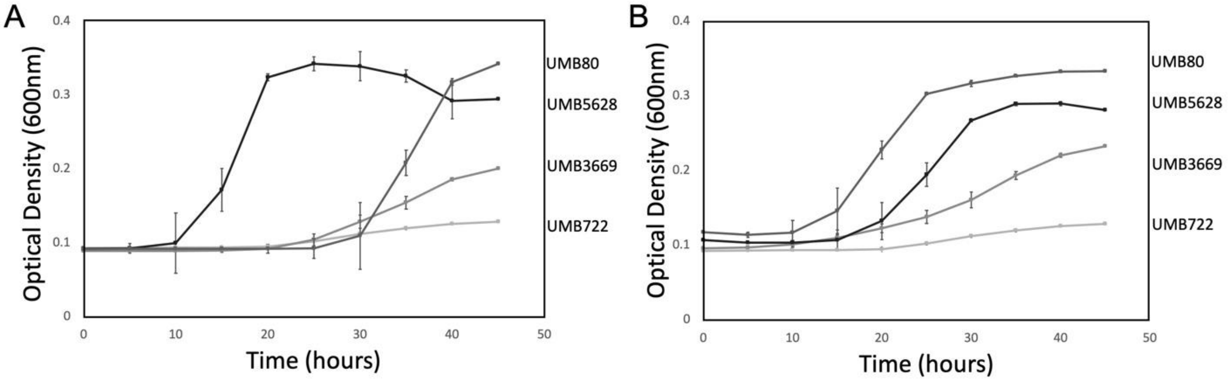

Fig. S4. Growth curves of ***A. urinae*** **in BHI media.** Comparison of growth patterns of strains during 48 h after inoculation into BHI media from BHI agar plates (A) and another 48 h after 1:10 passage of liquid culture into fresh BHI (B). Each time point is an average of four reads.

Biology Open • Supplementary information

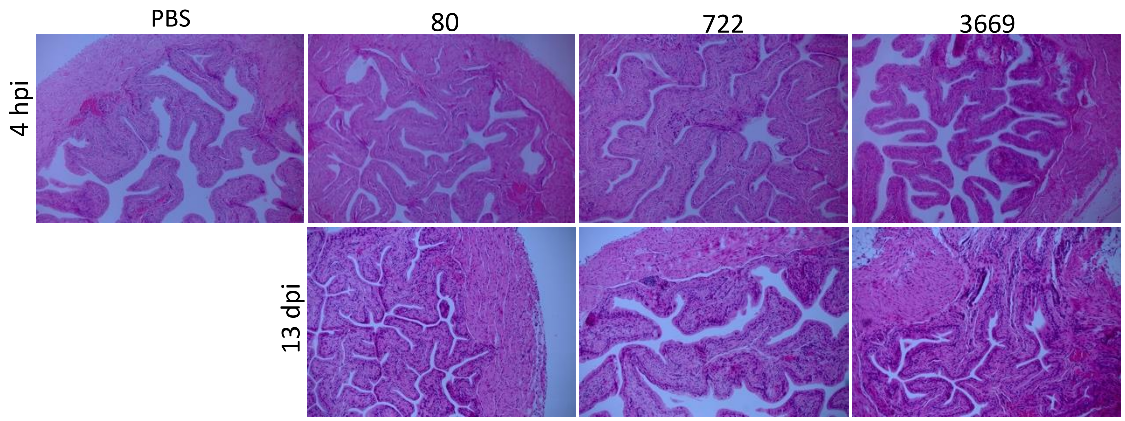

Supp

leme

ntary

Fig. S5. *A. urinae* does not cause exfoliation or inflammation in the bladders of C3H/HeN mice. Formalin-fixed, paraffin-embedded bladder sections collected from mice inoculated with the indicated *A. urinae* strains and sacrificed at 4 hpi (top row) or 13 dpi (bottom row), stained with hematoxylin and eosin.

Biology Open • Supplementary information

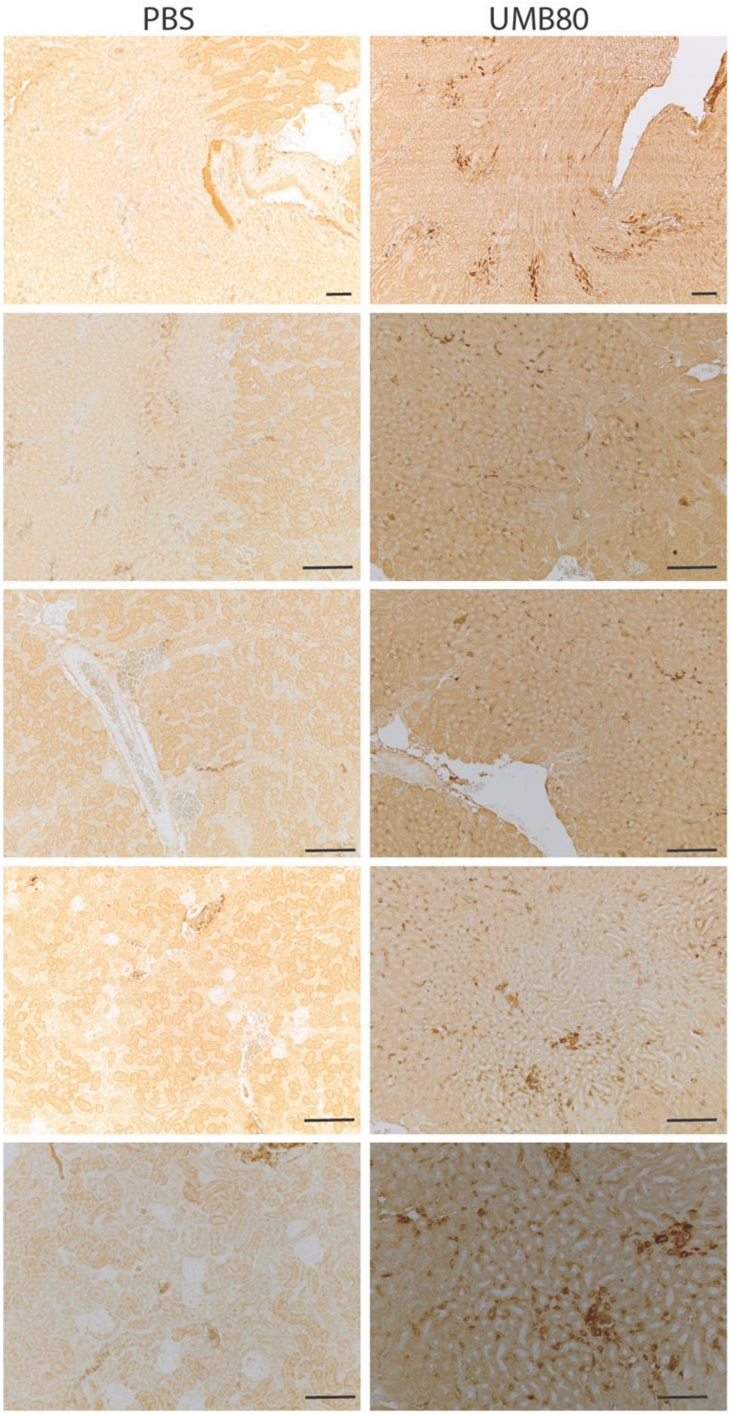

Fig. S6. Additional immunohistochemisty images of *A. urinae* in kidney tissue.

Formalin-fixed, paraffin-embedded kidney sections stained with anti-peptidoglycan antibody. Scale bars 100 μm.

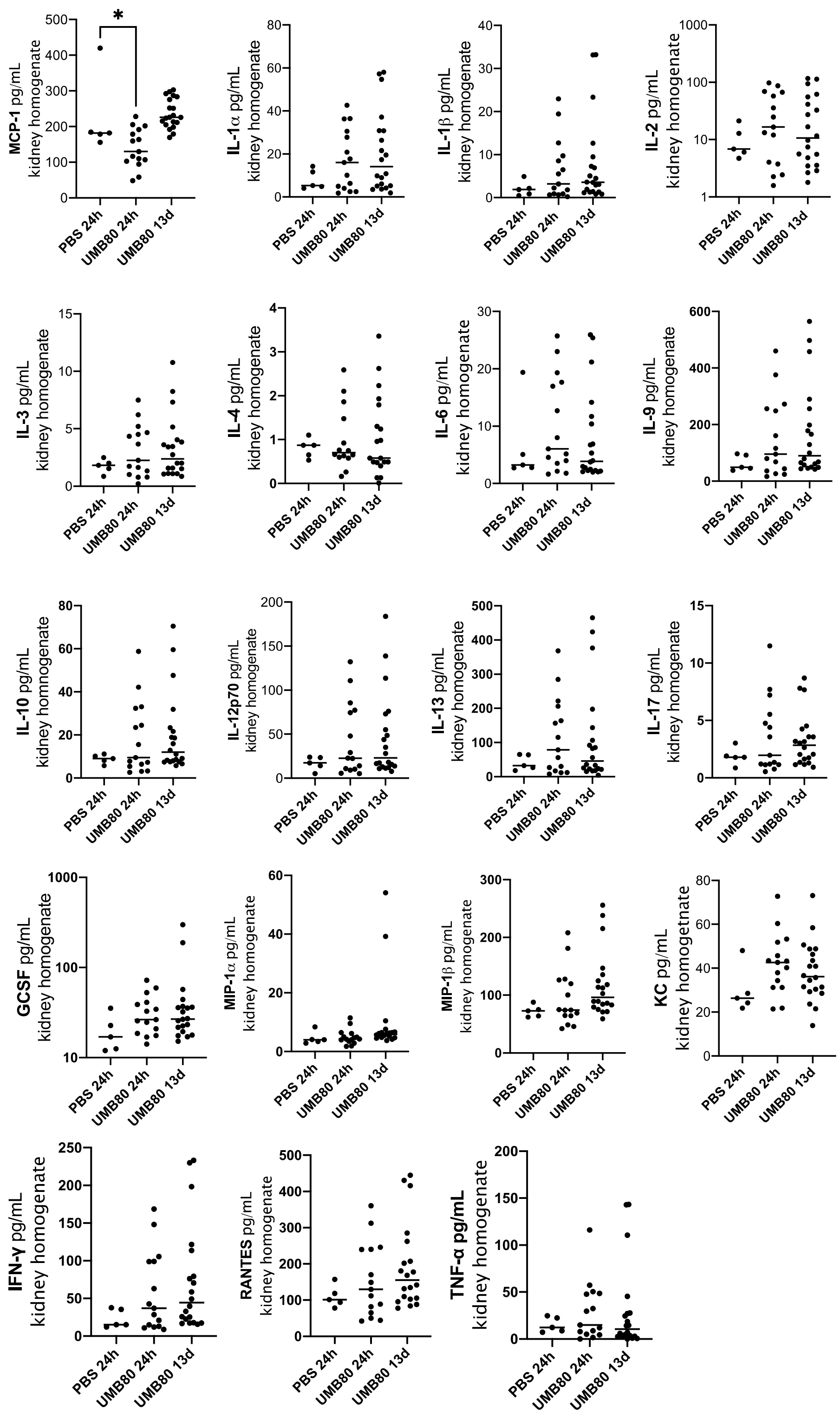

Fig. S7. Cytokine levels in kidneys. Kidney homogenate supernatants collected at the indicated timepoints from control (PBS) or UMB80-inoculated C3H/HeN mice.

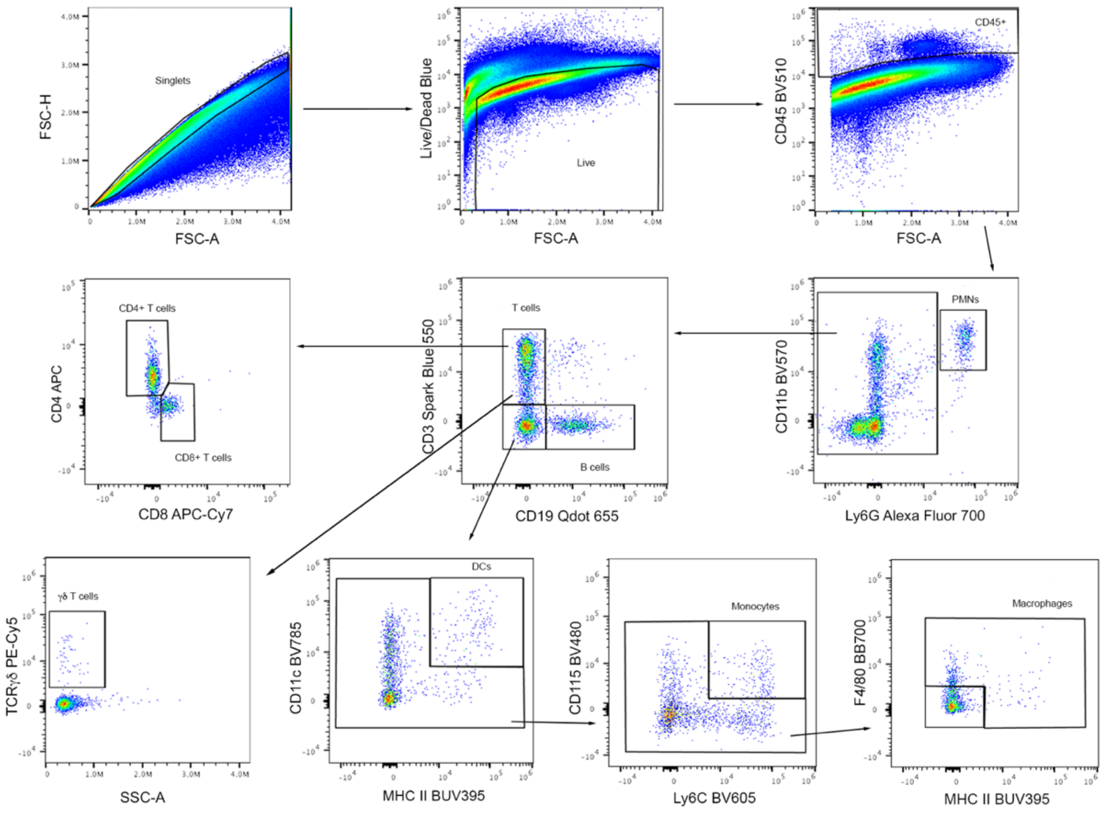

Fig. S8. Flow cytometry gating strategy. Kidney cells were stained with the indicated antibodies and immune cell populations determined as indicated.

Biology Open • Supplementary information

Table S1. Distribution of *A. urinae* strains in relation to clinical outcome

	Bac	IE	SUI	UTI	UUI	OAB	Control
A. urinae	7	8	1	8	0	0	1
A. urinae B	0	0	0	2	3	4	0
A. urinae C	2	0	2	5	5	1	0
A. urinae D	7	2	1	4	6	5	1

Biology Open • Supplementary information
